# Modular Biological Function Is Most Effectively Captured by Combining Molecular Interaction Data Types

**DOI:** 10.1371/journal.pone.0062670

**Published:** 2013-05-03

**Authors:** Ryan M. Ames, Jamie I. MacPherson, John W. Pinney, Simon C. Lovell, David L. Robertson

**Affiliations:** 1 Computational and Evolutionary Biology, Faculty of Life Sciences, The University of Manchester, Manchester, United Kingdom; 2 Centre for Integrative Systems Biology and Bioinformatics, Division of Molecular Biosciences, Imperial College London, London, United Kingdom; University Of Oxford, United Kingdom

## Abstract

Large-scale molecular interaction data sets have the potential to provide a comprehensive, system-wide understanding of biological function. Although individual molecules can be promiscuous in terms of their contribution to function, molecular functions emerge from the specific interactions of molecules giving rise to modular organisation. As functions often derive from a range of mechanisms, we demonstrate that they are best studied using networks derived from different sources. Implementing a graph partitioning algorithm we identify subnetworks in yeast protein-protein interaction (PPI), genetic interaction and gene co-regulation networks. Among these subnetworks we identify cohesive subgraphs that we expect to represent functional modules in the different data types. We demonstrate significant overlap between the subgraphs generated from the different data types and show these overlaps can represent related functions as represented by the Gene Ontology (GO). Next, we investigate the correspondence between our subgraphs and the Gene Ontology. This revealed varying degrees of coverage of the biological process, molecular function and cellular component ontologies, dependent on the data type. For example, subgraphs from the PPI show enrichment for 84%, 58% and 93% of annotated GO terms, respectively. Integrating the interaction data into a combined network increases the coverage of GO. Furthermore, the different annotation types of GO are not predominantly associated with one of the interaction data types. Collectively our results demonstrate that successful capture of functional relationships by network data depends on both the specific biological function being characterised and the type of network data being used. We identify functions that require integrated information to be accurately represented, demonstrating the limitations of individual data types. Combining interaction subnetworks across data types is therefore essential for fully understanding the complex and emergent nature of biological function.

## Introduction

Computational analysis of large-scale data sets is undoubtedly revealing an increasingly complete functional map of the cell [1]. In terms of molecular function, the field of Systems Biology is largely defined by a focus on interacting components. A level of importance set out by Hartwell and colleagues in their seminal article [2], which emphasised the modular nature of molecular function. In recent years networks have become the primary paradigm of representation of molecular interactions, reviewed in [3]. Usually functional modules and subnetworks are assumed to be one and the same, for example, a range of graph-property based approaches have been developed that identify subnetworks in protein-protein interaction [4], metabolomic [5], gene expression [6] and genetic interaction data sets [7]. However, these analyses potentially lead to an incomplete picture of function, since function usually arises from the coordinated and highly-specific operation of molecules of different types.

When studies do attempt to integrate distinct data sets, e.g.,[8]–[11], the emphasis has for the most part been placed on reconciling data-types, predicting gene function [12], [13] or identifying new interactions [14], as opposed to comprehensively delimiting the modules that comprise a specific unit of molecular function. For example, it has been reported that there is very limited overlap between genetic interaction and protein interaction data [7], despite both being clearly linked to molecular phenotype. Although it is clear that genetic interactions are best explained by considering epistasis within and between modules [5], [15], an integrated understanding of molecular and cellular function remains elusive.

Biological annotation, such as provided by Gene Ontology (GO) terms [16], are widely used to analyse functional characteristics of different data-types. This is, in part, because GO encapsulates and describes the modular nature of biology. Annotation enrichment methods for characterising protein or gene sets are widespread [17] and have also been used for determining the functions of subnetworks [18]. However, biological annotations are, necessarily, only a proxy for true function, derived from observable traits. Given the widespread use of biological annotation to characterise function, it is imperative to ascertain both the extent and reliability of networks constructed from different data to recapitulate biological function meaningfully. Importantly, such analysis should highlight the areas of biological function best described by each data type. Furthermore, by combining different data types in a single combined network we can determine whether a deeper biological insight can be gained from the integration of multiple data types.

Here we use a graph partitioning approach combined with annotation enrichment to identify how different interaction data-types capture functional modules at the molecular level using the well characterised yeast, *Saccharomyces cerevisiae*, as a model. Three interaction networks were constructed using: (i) protein, (ii) genetic and (iii) gene co-regulation interaction data. In addition, a combined network was created by integrating interactions from these networks. Each network was exhaustively partitioned to identify highly connected subnetworks, that form a set of subgraphs. Based on reciprocal best hits we identify subgraphs with significant overlap between the different data types and show these overlaps can represent related functions as represented by the GO. We next investigated the relationship between the subgraphs from the different data types (and a combined network) and GO. Our results show striking differences in both the ability of networks derived from different data types to capture specific functional modules and also in the total functional space that is covered by each network. By integrating subgraphs from different networks to form new composite subnetworks, we identify more comprehensively the components of functional modules, i.e., cohesive groupings of molecules not fully defined by a single data type.

## Results

### Interaction Networks and Subgraph Identification

Four interaction networks were assembled from large-scale *S. cerevisiae* data: a protein-protein interaction (PPI) network consisting of 12,182 interactions between 3,339 genes, a genetic interaction network consisting of 42,546 interactions between 3,529 genes, a co-regulation network consisting of 3,006,725 weighted interactions between 4,358 genes, and a combined network consisting of 3,052,053 unique weighted edges between 5,489 genes (Files S1–S4). Individual subgraphs isolated from connected subnetworks identified by graph partitioning, were produced for all these interaction networks (Figure S1). A total of 9,590, 9,227, 12,889 and 11,383 unique subgraphs were obtained from the PPI, genetic, co-regulation and combined interaction networks, respectively. These subgraphs include between 3 and 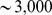 genes. The set of subgraphs forms a sample of the possible subnetworks at every level of granularity, hence providing an efficient basis for studying the functional organisation of the interactome from the most general to the most specific. As this method allows genes to appear in more than one subgraph from a network, we checked how often genes recur in subgraphs. We find that genes only appear in at most ∼1% of subgraphs, due to our subgraph validation and removal of identical subgraphs.

In order to validate the subgraphs we investigated their edge density, which is a measure of the number of links relative to the number of nodes present in a subgraph (Figure S2). The rationale is that biologically meaningful subgraphs will be more cohesive, i.e., there will be more interactions between nodes within the subgraph relative to interactions between nodes from different subgraphs. Therefore we selected only those subgraphs with significantly more interactions between nodes within a subgraph than interactions between subgraphs. We see that in the PPI and genetic networks as the subgraphs get smaller, as the network is split into more partitions, the edge density of the subgraphs increases. In the case of the co-regulation and combined networks, we define a weighted density measure that decreases with increasing subgraph size and is clearly apparent for subgraphs with >∼40 genes (Figure S2). Collectively, this confirms that subgraphs of a range of sizes capture cohesive subgroups of interacting genes. We therefore surmise that this set of subgraphs, or integrated subgraphs from different networks, will correspond to biological modules.

### Congruent Network Subgraphs

**Figure 1:**
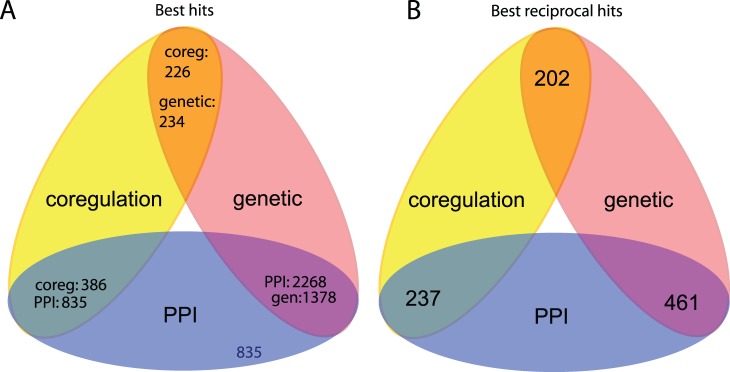
Results from best hits analysis. (A) Number of subgraphs from one network (named outside of the intersection) that are a best hit to a subgraph from another network (named within the intersection). (B) Number of best reciprocal hits between subgraphs from two networks.

In order to ascertain whether novel functional modules can be identified by the integration of data, we determined the extent to which subgraphs from the different data types are congruent. To do this we investigated whether the partitioning of different networks had resulted in the production of pairs of subgraphs from different networks that have significantly intersecting gene sets. We term such pairs “congruent subgraphs”. By comparing subgraphs from the PPI, genetic and co-regulation networks, we identified statistically significant gene intersections and subsequent “best hits” and “best reciprocal hits” between the subgraphs of two networks (see Methods for more details). A best hit represents a significant gene intersection between two subgraphs where one subgraph best matches the other, where best match is determined using the maximal Matthews correlation coefficient (MCC). A best reciprocal hit, again, represents a significant gene intersection, where both subgraphs are the best match to one another. Thus, best reciprocal hits indicate the strongest congruence between subgraphs from different networks. A summary of best hits and best reciprocal hits are given in Figure 1A and B, respectively.

**Figure 2:**
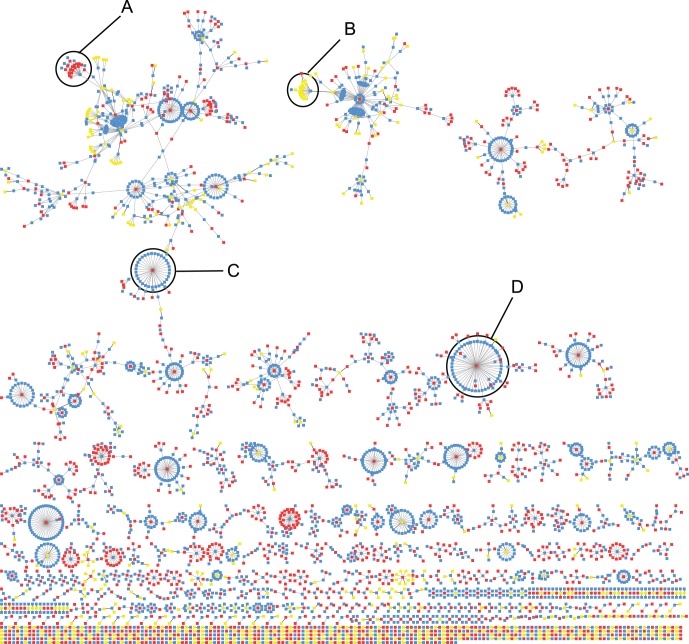
Network of best hits between subgraphs of PPI, genetic and coregulation networks. Nodes represent individual subgraphs with blue, red or yellow nodes corresponding to subgraphs from the PPI, genetic or co-regulation networks, respectively. Edges represent links between subgraphs with a statistically significant intersection of 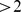 genes with an MCC 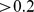. Only the best intersection between each network comparison, defined by MCC score, is shown. Letters A to D indicate high-degree neighbourhoods that consist of a node with degree 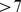 and all neighbours of that node.

To obtain a high-level insight into the congruence relationships between subgraphs from different networks, we visualised best hits (and best reciprocal hits) using a network, where nodes represent subgraphs and edges represent the hits (Figure 2). From a total of 4669 subgraphs that are involved in a best hit with one or more subgraphs, 3689 subgraphs are involved in a best hit with just one other subgraph, while a minority of subgraphs have many more best hits; the node degree fitting a power-law distribution. A repeated topological pattern of this best hits network (Figure 2) is for the subgraph of one network to be connected to a large number of subgraphs from one other network. Interestingly, there are 115 subgraphs that have a degree 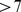 (top 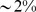). These subgraphs, that we refer to as *high-degree subgraphs*, are a set of genes that are repeatedly identified by partitioning networks into different sized partitions. Therefore, high-degree subgraphs and their hits appear to be robust sets of highly connected genes that transcend multiple networks. We hypothesised that high-degree subgraphs might have particular functional significance. Indeed, high-degree subgraphs and the subgraphs that are their best hits (together termed *high-degree neighbourhoods*) are: (i) significantly more likely to be enriched for one or more GO terms and (ii) capture GO functions with significantly better accuracy than subgraphs that are not congruent, in all networks (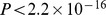, two-tailed Mann Whitney U test, in all cases), collectively indicating that the congruent subgraphs are more likely to be real functional modules. Furthermore this result highlights the value of integrating information between networks in order to validate network subgraphs.

To further investigate the usefulness of combining network data, we devised a method for testing whether new, biologically relevant functional links can be made by merging strongly congruent subgraphs. Subgraphs from all networks are frequently enriched for multiple biological functions (Table 1), we term this co-enrichment. Interestingly, many pairs of GO terms are co-enriched in each network, including pairs from the same and different ontologies and also from both related (descendent or ascendent) and unrelated GO terms from the same ontology (Table 2). New co-enriched GO term pairs are produced by merging best reciprocal hits from each network combination (Table 1). These new pairs represent biological functions that include a common subnetwork but are only co-enriched in networks that comprise interaction data from more than one source.

**Table 1:** GO term enrichment among congruent subgraphs.

Source network	Subgraphs	Number	Mean no. ofenriched GO terms	Mean max MCCper subgraph
PPI	high-degree neighbourhoods	2801	135.2	0.509
	all	9591	101.4	0.468
Genetic	high-degree neighbourhoods	1567	81.4	0.256
	all	9228	24.9	0.157
Co-regulation	high-degree neighbourhoods	601	15.4	0.138
	all	12889	7.1	0.095

**Table 2:** GO terms that are co-enriched in network subgraphs.

		Co-enriched GO term pairs			
Source	Network	All	Same ontology,related	Same ontology,unrelated	Differentontology
Whole network	PPI	3910331	1.1%	41.8%	57.0%
	Genetic	1035343	3.0%	41.2%	55.8%
	Co-regulation	224188	6.6%	39.2%	54.3%
Best reciprocal hits	PPI vs. Genetic	56154	0.3%	38.5%	61.3%
	PPI vs. Co-reg	17201	0.2%	34.4%	65.5%
	Genetic vs. Co-reg	3817	0.03%	41.2%	58.8%

**Figure 3:**
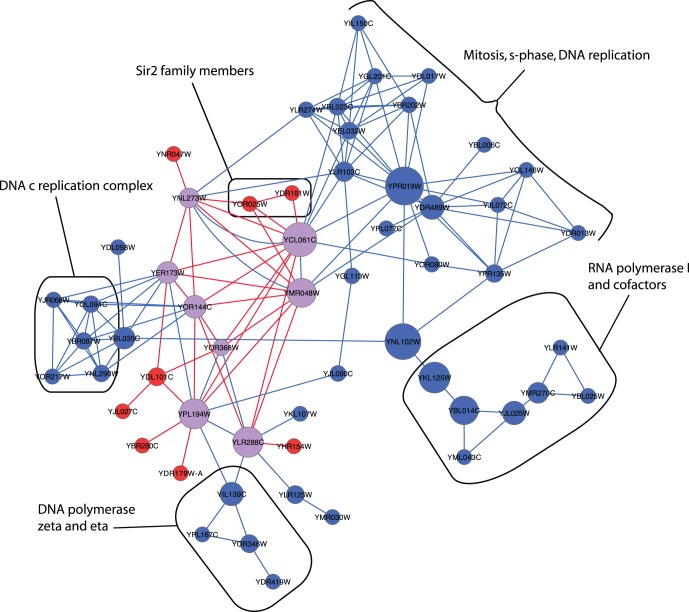
A subnetwork that represents merged subgraphs that are best reciprocal hits. This functional module characterises control of DNA replication. Nodes represent coding genes. Blue nodes genes from a PPI network subgraph, red nodes represent nodes from a genetic network subgraph. Nodes shown in purple represent genes that are present in both the PPI and genetic subgraphs. Node diameter is proportional to the node betweenness. Blue edges represent PPIs between encoded proteins and red edges represent genetic interactions between genes.

To illustrate, Figure 3 shows a PPI and a genetic subgraph that are best reciprocal hits, merged into a subnetwork. Several of the nodes identified by both subgraphs are clearly highly central to this subnetwork and have high node betweenness coefficients, e.g, YCL061C (MRC1), YMR048W (CSM3), YLR288C (MEC3) and YPL194W (DDC1). Furthermore several genetic interactions between these central genes also have high edge betweenness coefficients. Individually, both subgraphs are significantly enriched for genes involved in DNA replication and cell cycle control (GO:0006260 and GO:0007049). However, by combining these two subgraphs 81 new functional links are made between GO terms that are not co-enriched in subgraphs from any single network but are co-enriched when subgraphs from different networks are combined. Specifically, presence of Sir2 family genes (YOR025W and YDR191W) that are NAD(+)-dependent histone deacetylases involved in cell cycle progression [19] cause the new links, such as linking NAD binding (GO:0070403) to S phase of mitotic cell cycle (GO:0000084) and DNA replication factor C complex (GO:0005663). The Sir2 family members genetically interact with several proteins that are central to the subnetwork, including YCL061C (S-phase checkpoint protein) and YMR048W (replication fork associated factor). Hence, by combining network data-types novel and biologically meaningful functional links can be identified.

### Gene Ontology Coverage by Subgraphs Derived from Different Data Sources

To further investigate the range of biological functions that are captured by network subgraphs we looked for enriched GO terms in the subgraphs from all networks, including a combined network. Many network subgraphs consist of gene sets that are enriched for specific biological functions. Table 3 summarises functional enrichment for subgraphs from each network, for each of the three ontologies: biological process, molecular function and cellular component. We use the percentage of all annotated terms that are enriched terms in subgraphs to represent the coverage of a particular ontology. We see that the PPI network captures functional annotations with the greatest coverage for the cellular component ontology (93%), compared to subgraphs from the genetic and co-regulation networks (Table 3). The biological process ontology is covered about the same by the different data sources (82–83%). Interestingly, we find that the greatest coverage (over 92%) for all three ontologies is captured using a combination of data sources. MCCs, used to measure the accuracy with which subgraphs capture specific GO term annotations, were significantly different for enriched terms for different networks across all three ontologies (Kruskal-Wallis rank sum, PPI, Genetic and co-regulation all P<2.2×10^−16^). Combined these results indicate that the distinct network data sources have significantly different abilities to capture the biological functions represented by GO.

**Table 3:** GO coverage for each network.

WholeNetworks		PPI (HT)^a^	Genetic	Co-regulation	Combined
BP	Enriched terms	2271 (2547)	2265	2169	2639
	Total terms	2710 (3268)	2736	2655	2694
	Coverage (%)	84 (77)	83	82	98
	Average MCC	0.43 (0.10)	0.25	0.19	0.31
MF	Enriched terms	893 (1118)	1006	1012	1237
	Total terms	1541 (1913)	1457	1346	1333
	Coverage (%)	58 (58)	69	75	93
	Average MCC	0.42 (0.10)	0.25	0.22	0.29
CC	Enriched terms	682 (691)	558	556	651
	Total terms	660 (756)	632	629	713
	Coverage (%)	93 (91)	80	80	97
	Average MCC	0.60 (0.12)	0.26	0.19	0.45
**Common gene** **Networks**		**PPI**	**Genetic**	**Co-regulation**	**Combined**
BP	Enriched terms	1094	1334	191	1461
	Total terms	1778	2056	2056	2056
	Coverage (%)	61	64	9	71
	Average MCC	0.21	0.20	0.25	0.20
MF	Enriched terms	302	356	53	446
	Total terms	666	818	818	818
	Coverage (%)	45	43	6	54
	Average MCC	0.24	0.23	0.20	0.22
CC	Enriched terms	282	340	39	368
	Total terms	413	508	508	508
	Coverage (%)	68	66	7	72
	Average MCC	0.25	0.19	0.25	0.21

a HT refers to enriched terms found in subgraphs from the high-throughput PPI network.

To ensure that it is the structure of these networks that is responsible for the identification of enriched terms in subgraphs, we randomised the GO annotations in each network. Unsurprisingly, we see little enrichment in these randomised subgraphs. Subgraphs from the PPI network show the most enrichment with 17% of terms from the cellular component ontology enriched. Although this may seem high it is a large reduction in the coverage of the cellular component ontology compared to the original analysis (Table 3). In all other networks and ontologies the coverage of GO is reduced to ≤10%. This demonstrates that it is the structure of these networks that holds the functional information and partitioning these networks results in subgraphs that represent real functions.

Our high-confidence PPI network may be prone to ascertainment bias because we only select interactions that have been reported more than once. This may favour interactions between genes that have been extensively studied. To control for this ascertainment bias we repeated the enrichment analysis using only data from high throughput experiments. We see that the coverage of the biological process ontology has been lowered from 84% to 77%, whereas the cellular component ontology is only slightly affected and the coverage of the molecular function ontology remains the same (Table 3). Overall, we conclude that using a high-throughput PPI network does not affect the trends of our results.

In order to investigate the non-independence of GO relative to the network data types, we investigated the coverage of GO but this time using the different annotation types: inferred electronic annotations (IEAs) and non IEAs. We find that in both cases the overall coverage of GO is reduced for all data types (Table 4). Interestingly, when we use a subset of GO, the combined network no longer shows any greater coverage than the individual data types. Additionally, we also looked at terms enriched only in subgraphs from the combined network and identified the annotation types associated with these terms. We see that the most frequent annotation types are experimental (mutant phenotype, direct assay and genetic interaction), computational (sequence or structural similarity) and IEAs. These results suggest that no single network is overly annotated from (or used to annotate) a single annotation type and it is only when we use the entirety of GO that we see the improved performance of the combined network.

**Table 4:** GO coverage for networks without inferred electronic annotations and using only inferred electronic annotation.

No IEA		PPI	Genetic	Co-regulation	Combined
BP	Enriched terms	2035	2027	1942	2396
	Total terms	2744	2714	3030	3172
	Coverage (%)	74	74	64	75
	Average MCC	0.14	0.13	0.12	0.11
MF	Enriched terms	690	749	778	960
	Total terms	1226	1401	1665	1777
	Coverage (%)	56	53	46	54
	Average MCC	0.16	0.14	0.13	0.12
CC	Enriched terms	629	495	504	662
	Total terms	682	591	700	720
	Coverage (%)	92	83	72	91
	Average MCC	0.18	0.13	0.11	0.14
**Only IEA**		**PPI**	**Genetic**	**Co-regulation**	**Combined**
BP	Enriched terms	695	713	751	861
	Total terms	996	991	1147	1186
	Coverage (%)	69	71	65	72
	Average MCC	0.15	0.14	0.12	0.12
MF	Enriched terms	475	505	562	664
	Total terms	887	981	1184	1261
	Coverage (%)	53	51	47	52
	Average MCC	0.16	0.14	0.14	0.13
CC	Enriched terms	215	176	163	220
	Total terms	298	251	299	320
	Coverage (%)	72	70	54	68
	Average MCC	0.15	0.13	0.10	0.12

Interestingly, not all identified subgraphs are enriched for GO terms. Indeed, there are 28, 1,797, 5,166 and 3,848 subgraphs with no enrichment in the PPI, genetic, co-regulation and combined networks respectively. Interestingly, there are very few unknown subgraphs in the PPI network, whereas there are many in the co-regulation network. These unknown subgraphs may be erroneous localities within the networks, they may be subgraphs that represent real functions but the members are poorly annotated or they may represent functions not well described by the GO. Dutkowski *et al.* [20] have recently identified groups of genes in yeast that represent novel ontology terms not included in the GO. This result suggests that the unknown subgraphs identified in this study may represent real functional modules not accurately described by GO.

To ensure that the improved characterisation of biological function of the combined network was not an artefact of the network having the most nodes, we repeated the enrichment analysis with the common gene networks. Here, the only difference between the networks are the edges as each network contains the same nodes. We see that the coverage of GO is reduced in all networks, likely because the networks all have fewer nodes (Table 3). However, this trend does not affect all the networks equally as the co-regulation network has a severe reduction of GO coverage. This is likely to be caused by the different reduction of edges in these networks. Both the PPI and genetic networks retain 11 and 16% of edges in the common network respectively, whereas the co-regulation network only retains around 4%. Interestingly, we find that the combined network assembled from common nodes still has the greatest coverage of GO for all three ontologies, despite only containing 4% of edges from the original network. Furthermore, there are 156 enriched terms from the combined network subgraphs that are not enriched in the subgraph from any other network. Therefore, we conclude that the results presented for the original combined network are not affected by the number of nodes in the network and the combination of information from a variety of sources allows for the identification of areas of biological function not found by inspecting these networks individually.

### Accuracy of Gene Ontology Term Enrichment by Network Subgraphs

GO terms can refer to very common functions (i.e, be assigned to a considerable fraction of all genes), or refer to specialist functions (i.e., be assigned to very few genes), or lie somewhere between these two extremes. In order to ascertain whether the functional categories enriched in subgraphs from different networks were biased towards capturing general (versus more specialist) functions, we looked at the number of subgraphs enriched for generalist and specialist terms and the accuracy of this enrichment (Figure S3). We find that PPI subgraphs and subgraphs from the combined network capture functions with relatively less bias for specialist or general terms than either the genetic or co-regulation subgraphs; the genetic network subgraphs displaying a bias for capturing less specific functions at the expense of highly specialist functions, whereas an approximately opposite trait can be observed for co-regulation network subgraphs. It is clear that very general functions with a membership of over ∼100 genes are difficult to capture from any of the networks, relative to more specialist functions with fewer members. Furthermore, the accuracy with which the function is captured diminishes when the function is defined by many genes.

**Figure 4:**
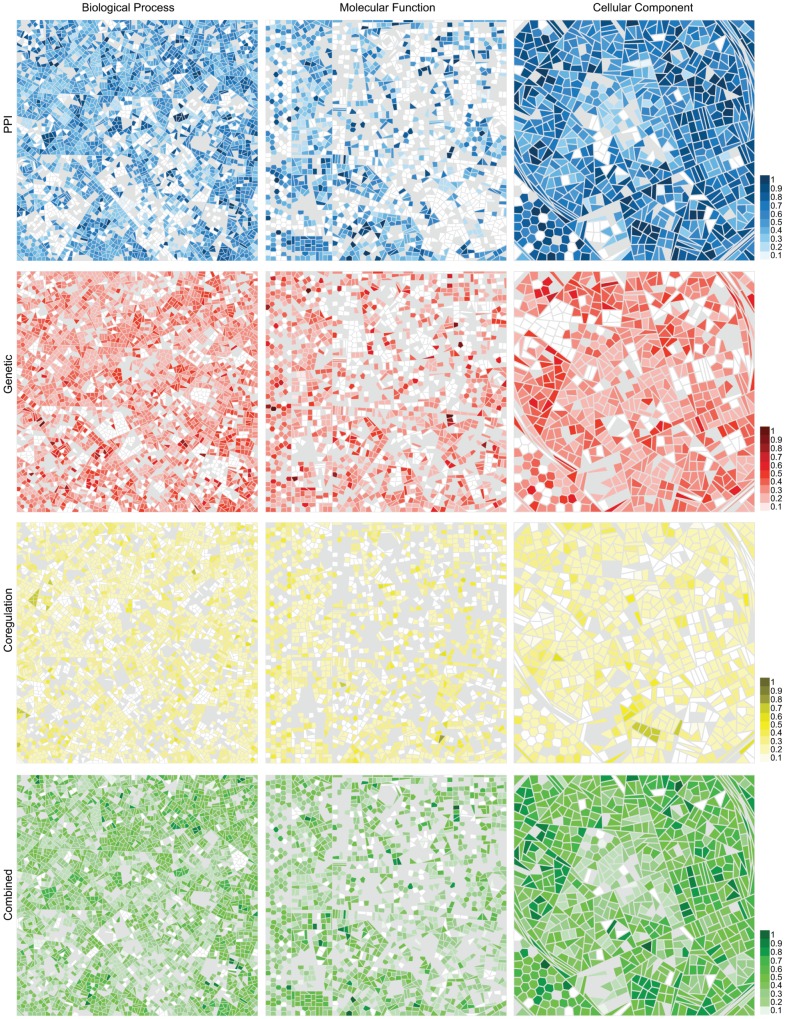
GLASS visualisation of enriched GO terms. Each cell represents a GO term and is coloured blue, red, yellow or green if one or more subgraphs are enriched for that GO term in the PPI, genetic, co-regulation or combined networks, respectively. The intensity of each coloured cell shows the best MCC of the subgraphs with enrichment for that term. Grey coloured cells are those GO terms which have only one or no associated genes in that network.

To further investigate the capture of distinct functional categories by network subgraphs, we visualised gene ontology terms using a Voronoi tree-mapping approach (Figure 4). In the tree maps, each cell represents a GO term, where terms of similar functions are grouped together. The maps from different networks are directly comparable with the equivalently positioned cells in each tile representing the same GO terms. The intensity of cell shading indicates the accuracy with which the GO term is captured by a network, using MCC score as the accuracy measure. Importantly, the Voronoi maps (Figure 4) highlight the disparity between the ability of network subgraphs to capture certain types of functional data. Cellular component annotation appears to be the easiest type of biological function to capture, using any type of network data. Conversely molecular function is more difficult to capture. This tree-mapping approach also highlights that certain functional areas within each ontology can be either successfully captured, or are difficult to capture, using the network data. In contrast, some areas are clearly shaded in all maps from the same ontology. The ability therefore of different networks to capture functional relationships, is related both to the type of data used to create the network, and also the specific function in question.

**Figure 5:**
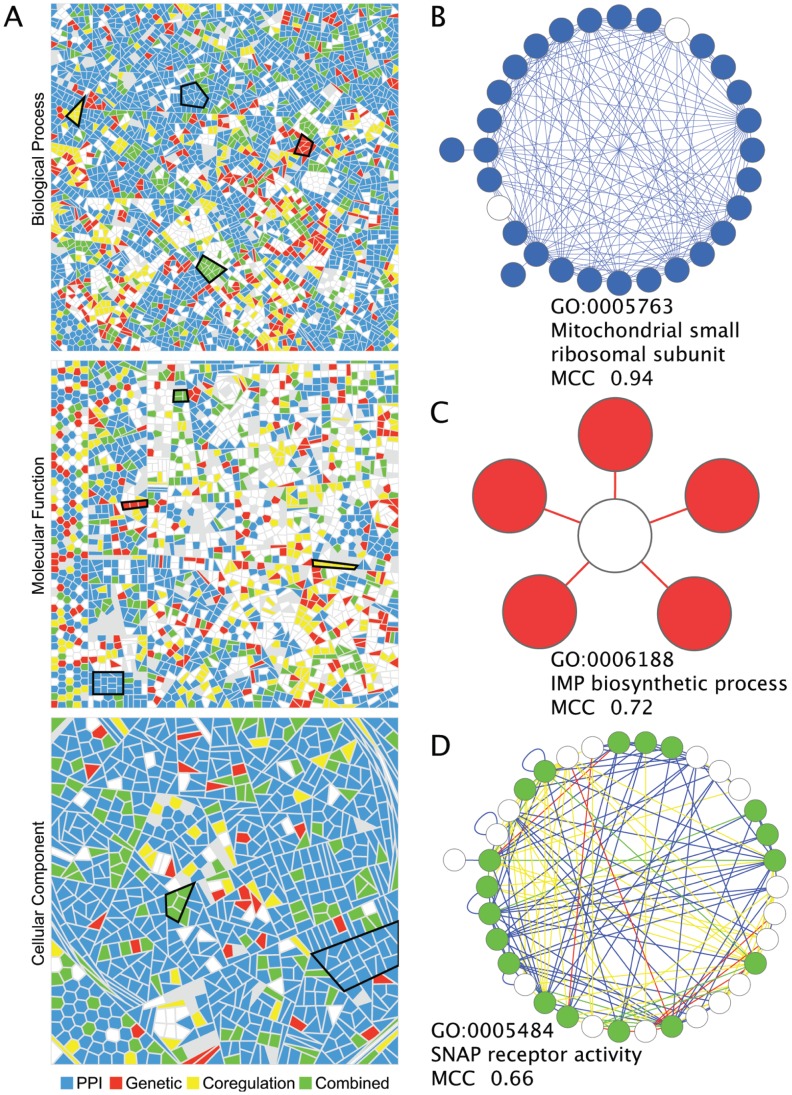
Composite functional maps. (A) GLASS visualisation in which each cell represents a GO term, coloured according to subgraphs that have the highest MCC for the enriched term. Blue, red, yellow and green colours indicate the subgraph with the highest MCC is from the PPI, genetic, co-regulation or combined network, respectively. Grey coloured cells are GO terms which have only one or no associated genes in any network. Areas ringed in black show examples of areas of the ontology which are best characterised by a single network. Panels B–C show examples of the best characterised subgraphs between all networks: (B) The mitochondrial small ribosomal subunit GO term is best represented by a subgraph from the PPI network. (C) A genetic subgraph best represents the IMP biosynthetic process GO term. (D) The GO term, SNAP receptor activity, is best represented by a subgraph in the combined network, created from all nodes and edges in the PPI, genetic and co-regulation networks. Nodes are coloured blue, red or green if they are present in the PPI, genetic or combined network, respectively, and are associated with each enriched GO term. White nodes represent nodes in a subgraph that are not associated with the enriched GO term. Edges are coloured blue, red, yellow or green if they are present in the PPI, genetic, co-regulation or combined network, respectively.

Creation of a composite tree-map (Figure 5A), where the cell colours represent the network from which the terms are most accurately captured, allows direct comparison. From the trees on which the maps are based, we can identify distinct areas within an ontology, that are best characterised by subgraphs from a single network. Examples of such areas are outlined in Figure 5A. We can identify specific subgraphs from a single network that accurately characterise a single GO term. In the PPI network a single subgraph represents the mitochondrial small ribosomal subunit cellular component term, where 28/30 members annotated with the term and a MCC of 0.94 (Figure 5B). From the genetic network we have identified a subgraph that accurately represents the Inosine monophosphate (IMP) biosynthetic pathway and enzymes representative of the purine biosynthesis pathway (Figure 5C). These findings demonstrate that different areas of biological function are best represented by different types of biological data.

Interestingly, all three ontologies have areas that are best represented by subgraphs from the combined network. Therefore, the combining data-types from multiple biological networks can improve characterisation of certain biological functions, when compared to analysis of any network in isolation. Indeed, terms from the molecular function ontology are enriched with the greatest coverage by the subgraphs from the combined network (Table 3). To give a specific example, the synaptosomal-associated protein (SNAP) receptor activity molecular function term is best represented by a combined network subgraph which incorporates edges from all other networks (Figure 5D). We have included lists of the 20 most accurately represented GO terms from each network and ontology in File S5. Ultimately, it seems that although these networks capture overlapping areas of biology, there are functional modules that are most accurately characterised by a single network. Therefore, we find that the choice of data used to generate a network can have a significant effect on the ability of that network to answer specific biological questions.

## Discussion

We have shown that the integration of subgraphs from individual networks can reveal new functional groupings. Many of the subgraphs identified from the individual data-type networks have significant overlaps (Figure 1). Indeed, we find that some subgraphs are the best hits of many other subgraphs and these appear to capture GO functions very accurately (Figure 2). By merging subgraphs from the PPI and genetic networks that are best reciprocal hits to create a new subnetwork, we find we can identify new cohesive modules that describe a particular biological function better than any subgraph from individual networks (Figure 3). Collectively, these results show that not only can the integration of data reveal more functional information, but also the integration of individual modules from these data can reveal novel functional links.

Not surprisingly, we find different interaction data types capture different types of biological event, which can have varying degrees of contribution to a specific function. Moreover, we find differences in the characterisation of general and specialist GO terms between the networks (Figure S3). Therefore, it appears as though subgraphs from particular networks are better suited to capturing GO terms annotated by many genes whereas subgraphs from other networks better characterise GO terms annotated by few genes. From the three networks containing a uniform type of data, biological functions are most accurately and completely captured by the PPI network (Table 3 and Figure 5). In contrast, the molecular function ontology, which denotes enzymatic and biochemical properties of gene products, is only partially captured by PPI interactions, indicating that subgraphs that represent physical components and protein complexes contain both biochemically similar or unrelated subunits. More remarkable, however, is the almost complete extent to which PPI subgraphs captured functional annotations from both the cellular component (93%) and biological process (84%) ontologies (Table 3). The co-regulation network performed least effectively overall at capturing biological functions. This is potentially an unavoidable feature of gene expression data. As a measure of transcript abundance, gene expression data can only provide an estimate for the relative change at the level of the protein. Despite these drawbacks, the co-regulation network captures the majority of functions embodied by biological process and cellular component GO ontologies. Interestingly, the co-regulation network captures more molecular function GO terms than either the PPI or genetic networks (Table 3).

Genetic interactions imply a functional relationship between genes. Undeniably, these relationships have functional relevance from a organismal perspective, as they are expressed phenotypically. In a systematic study to characterise such within- and between-pathway genetic interactions in *S.cerevisiae*, Kelley and Ideker [9] identified that between-pathway relationships have a tendency to be better explanations for genetic interactions than within-pathway interactions. Here, the so-called “pathways” are cohesive subnetworks of proteins in the PPI network. Kelley and Ideker [9] classify genetic interactions as within-pathway or between-pathway, the former indicating a genetic interaction between elements of the same subnetwork and the latter indicating a genetic interaction between elements from a separate subnetwork. Thus, within-pathway genetic interactions are indicative of a functional PPI subnetwork, such as a protein complex. Furthermore, they identify that many between-pathway interactions link interdependent functional relationships. Between-pathway interactions thus indicate distinct functional PPI modules that collectively are essential units of a single, greater functional process. If these findings are upheld by GO annotation, genes involved in genetic interactions should always both be attributed with a given process or component annotation that captures their common cellular activity, be that either a relatively specialist or a very general function. Indeed, we show that 83% of the biological process terms and 80% of cellular component terms are captured by genetic interactions. Therefore, the interaction network derived from genetic interactions is clearly a reasonable choice of data for capturing physically interacting and process-related functions.

Our combined network differs from those of Kelley and Ideker [9], in that it is a weighted union of the PPI, genetic and co-regulation networks permitting direct comparison with the networks derived from a uniform data-type. Thus, it represents a union of both within- and between-pathway interactions. We find that in many cases this integrated view offers the most useful view of modular function (Figures 3 & 5). Moreover, the most notable aspect of the combined network is that the coverage of captured annotations is almost complete for each GO ontology. Molecular function GO annotations are more successfully depicted by combined data than by any other network we investigated (Table 3). These findings are true of networks assembled from a common set of nodes, controlling for network size (Table 3). Additionally, subgraphs from the combined network assembled from common genes were enriched for 156 terms that were not enriched by subgraphs from any individual network. This suggests that the combined network is more than the sum of the three individual networks and can identify areas of biological function that are not represented in the individual networks. However, the accuracy with which these functions are captured is generally not as great as for the PPI network (Table 3). This is perhaps due to a greater level of noise in the combined network compared to the PPI network, stemming from the co-regulation and genetic interaction data. Yet it is conceivable that a more refined data integration method, involving, for example, machine-learning of real functional links, could attenuate the error rate. Clearly, by continually adding more interaction data to biological models, we will inevitably capture additional functional links. Importantly, while we have demonstrated that each one of the three frequently studied types of biological interaction - PPI, genetic and gene co-regulation - all make a valid yet distinctive contribution to a network model, combined they reveal more about the modular nature of biological function.

It is unlikely that the networks used in this study represent the complete networks in yeast. Indeed, the genetic interaction network is built from experiments on only a subset of yeast genes [7] and all networks contain fewer genes than the ∼6000 annotated yeast genes. Therefore the improved coverage of GO and unique functional links identified from the combination of data suggests that this approach may be useful in organisms with incomplete interaction networks. Additionally, the identification of subgraphs with no known annotation is analogous to the novel terms identified in the network-extracted ontology (NeXO) [20]. As with uncharacterised terms present in NeXO, the unknown subgraphs in this analysis may represent true functional modules that are not in the GO. Hence, the characterisation of these unknown subgraphs may prove to be a useful step in expanding the GO to encompass as yet unknown functions.

In conclusion, our results show that a network derived from a single data-type is capable of defining certain areas of biological function with greater accuracy than networks from other sources. As a consequence the choice of interaction data directly influences the ability of networks to depict specific functional relationships. Certain networks are therefore better suited for studying specific biological functions. We also find that combined subnetwork data represents the greatest range of biological functions. Indeed, it appears as though the combination of interaction data may be able to characterise areas of biological function that cannot be characterised by a single network. In addition, our definition of both subgraphs derived from the combined network and congruent subgraphs are novel ways of identifying functional modules; specifically their strength is the definition of function that arises from concerted actions of diverse types of molecules and interactions. What these results collectively demonstrate is that a more complete perspective of a biological system is revealed by combining networks derived from multiple data-types. Interestingly, functional modules identified from congruent network subgraphs represent areas of biology that may only be understood through the combination of data of diverse types.

## Methods

### Network Generation

Four interaction networks were assembled where nodes represent genes and edges represent interactions between genes:

1. A PPI network was assembled with physical interaction data from the BioGRID database [21]. Interactions were only included in the network if there was evidence for that interaction from multiple sources. The PPI network therefore represents a high confidence set of physical interactions. As a control we also generated a low confidence PPI network created from only high throughput experimental evidence in order to minimise ascertainment bias.
2. A genetic network was built using data from [7], which was downloaded from the *Saccharomyces* Genome Database (SGD, www.yeastgenome.org). The network was built using a stringent P value cutoff for a genetic interaction of P<0.001.
3. A co-regulation network was built using expression profiling data from [22] where 300 separate treatments were performed and gene expression was recorded. Two genes were defined to be coregulated if they achieved a P value of 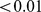 for expression using gene-specific error model from a single treatment. Gene nodes were connected by an edge if they were coregulated. Edges were weighted according to the frequency with which they are co-regulated across all treatments, where a greater weight denotes a greater frequency, defined by:

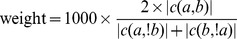



Where *a* and *b* are coregulated genes and 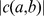 is the number of times *a* and *b* are coregulated over all treatments and 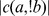 is the number of times *a* is coregulated but not with gene *b* over all treatments. For the purposes of subgraphing, weight values were rounded to the nearest whole number.

1. A combined network was created by pooling all data from the PPI, genetic and co-regulation networks. Edges from the different networks were weighted and weights were normalised so that the sum of edge weights contributed by each network was equal. Edges in the combined network were assigned a weight equal to the sum of weights for that edge in all contributing networks.

Additionally, a common gene network was assembled for each of the PPI, genetic, co-regulation and combined networks. These networks were made in order to control for the differences in gene content between the networks described above. In these networks only the edges differ between a common set of nodes. The networks were constructed by first selecting only those nodes present in all networks and secondly, ensuring that these nodes were connected within the network.

Note, genes were only included in the networks if they corresponded to an open reading frame in SGD.

### Subgraph Generation

Subnetworks were generated from networks using a *k*-way graph partitioning algorithm, kmetis [23]; see Figure S1. For a network 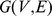, with *V* nodes and *E* edges, kmetis aims to partition nodes in to *k* sets of approximately equal size and minimise the number of edges that connect node sets. Resulting node-sets and the edges that link those nodes comprise a subnetwork. For a given network we identified the set of average node-set sizes *S* for every given partition that could be obtained using *k*-way partitioning where 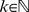 and 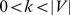. For all 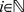 where 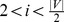 we selected 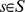 nearest in value to *i* and recorded the value for *k* corresponding to *s*. We performed *k*-way partitioning on the network using all distinct recorded values of *k*.

To identify subgraphs within the subnetworks produced by partitioning, we selected non-redundant largest-connected-components that contained more than two nodes. As Kmetis will always partition the whole graph in to *k* parts, it is likely that some of the subgraphs we produce do not represent *bona fide* localities within the network. Therefore, subgraphs were scored based on comparison between mean internal path length and path lengths to other subgraphs from the same partition. Specifically, the path length between all nodes was calculated using the Dijkstra method [24]. The mean intra-subgraph path length for all nodes was computed for all subgraphs and mean inter-subgraph path lengths were computed between every pair of subgraphs from the same partition. Following this, a one-tailed/one-sample t-test was used to ascertain whether the mean intra-subgraph path length is significantly smaller than the mean inter-subgraph path lengths, for a given subgraph. This check essentially ensures that kmetis has identified a *bona fide* location in the network and that the subnetwork is not simply a bi-product of the number of partitions made by the program. Any subgraph that did not achieve a P value of 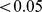 was discarded.

The edge density, *d*, for a subgraph, with *e* edges and *n* nodes, from an unweighted network was defined as the proportion of all possible gene-gene interactions that are present, calculated by:
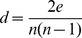



Similarly, weighted density, *h*, for a subgraph, with sum edge weight *w* edges and *n* nodes, from a weighted network that has mean edge weight 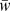, was defined as:
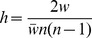



### Identification of Congruent Network Subgraphs

Subgraphs from different networks (excluding the combined network) were cross-referenced against one another and the statistical significance of the intersection in genes between two subgraphs was calculated by Fisher’s exact test. Here, the union of the two subgraphs formed our population, and our population successes were simply the genes in a single subgraph. We then treated the other subgraph as a sample from the population and our sample successes were the intersecting genes between the two subgraphs. To limit the number of comparisons, two subgraphs were only compared when the size of the two gene sets was not greater than ten-fold different. Matthews’s correlation coefficient (MCC) [25] values were calculated to quantify the precision and accuracy of the subgraph intersection using the formula:
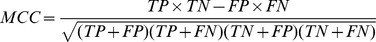



Where the true positives (*TP*) were the intersection between the two subgraphs. True negatives (*TN*) were the union of the subgraphs minus the intersection. Finally, our false positives (*FP*) and false negatives (*FN*) were the size of each subgraph respectively minus the size of the intersection. To reduce the number of statistical tests performed, P values were only calculated if the intersect between subgraphs (true positives) was 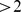 genes and the MCC was 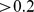. Resulting P values were corrected for having performed multiple tests [26]. For each subgraph from a network that had at least one statistically significant intersection (corrected P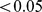) with subgraphs from another network a “best hit” was assigned to the subgraph intersection with greatest MCC score. Reciprocal best hits were defined as two best hits between subgraphs from different networks.

Note, the PPI data is a from a compendium of experimentally validated PPIs, whereas the other data sources are extrapolated from high-throughput experiments. Therefore, the quality of the derived networks, in terms of type I and type II error rates, are unlikely to be equivalent. Hence, direct comparison of the performance of each network at capturing aspects of biological function will undoubtedly not only reflect the information available from the type of interaction but also the error rate.

### Functional Enrichment in Network Subgraphs

We assigned function to identified subgraphs using the Gene Ontology (GO) [16]. GO annotation was retrieved from the GO download site. We used Fisher’s exact test to identify overrepresented GO terms for each subgraph. Here, our population set was all the genes present in the network and the number of genes in the network annotated with a particular GO term represented the population successes. We then treated each subgraph as a sample from the network and the subgraph genes annotated with the term as the sample successes. All *P* values were false discovery rate corrected using the method described in [26] with a significance cutoff of *P*<0.05. Additionally, we used the MCC as a measure of accuracy of our subgraphs for each overrepresented term. MCC was calculated by the formula described above. The true positives are the number of genes in a subnetwork annotated with the overrepresented GO term. The true negatives are the number of genes not in the subnetwork and not annotated with the GO term. False positives are the number of genes present in the subnetwork and not annotated with the term. Finally, the false negatives are the number of genes not in the subnetwork but are annotated with the overrepresented GO term.

We have also aimed to control for the potential confounding factors such as the non-independence of these networks and the GO. For example if PPIs are used to annotate the interacting genes with GO terms, the data may be biased such that the most highly connected genes are the most well annotated. We have attempted to control for confounding factors by repeating our above analysis after removing GO terms that have been inferred by electronic annotation, as these annotations are likely the result of high throughput experiments potentially containing errors. We also repeated the enrichment analysis using randomising GO term annotations within the network. Here, GO annotations were randomly assigned to genes, ensuring that the number of GO annotations and connectedness of genes remained the same.

Relative enrichment of GO terms, with respect to the number of genes represented by a term, was calculated for subgraphs from each network. First terms were binned according to the number of genes they represent in the network data set and the proportion represented by each bin was calculated. Next the same process was carried out for enriched terms represented by subgraphs with MCC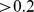. Enrichment was defined as the proportion for enriched terms minus the proportion for all terms, for each bin. Hence, enrichment values across all bins sum to exactly one.

In order to visually compare network coverage, semantic similarity (Lord et al., 2003) was used to determine the functional distance between genes and a tree-structure generated using neighbor-joining and represented in two dimensions using Voronoi Treemaps (Balzer and Deussen 2005; Balzer et al. 2005), implemented with GLASS (available at http://www.bioinformatics.ic.ac.uk/glass/). In this visualisation each cell represents a GO term, whose location within the panel is determined by the semantic distance to all other terms. A cell is coloured if one or more subgraphs from a particular network display enrichment for that term. The intensity of the colour is determined by the MCC of that subgraph for the enriched term.

### Network Visualisation and Analysis

All network visualisations were produced using Cytoscape [27]. Edge and node betweenness coefficients were calculated using the NetworkAnalyzer Cytoscape plugin [28].

## Supporting Information

Figure S1 **Network partitioning methodology.** Interaction networks were partitioned by using *k*-way partitioning. *k* represents the number of partitions for the algorithm to produce. Given the number of nodes in the network and the number of partitions we can estimate the average size, *s*, of the subgraphs produced by partitioning. Many different values for *k* were used in order to produce an extensive set of partitions with a wide range of sizes. We partitioned each network until until the average size of the partitions was estimated to ∼3 nodes.
(EPS)

Figure S2 **Summary of network subgraphs showing plots of (i) subgraph size against subgraph frequency (panels A, C, E and G), and (ii) subgraph size against the top 95th percentile of clusters ordered by edge density (panels B, D, F and H) for PPI, genetic, co-regulation and combined interaction networks, respectively.**
(EPS)

Figure S3 **GO enrichment among network subgraphs.** Two types of graph are shown: (i) bar plots A, B, C and D show the relative level of enrichment of GO terms pertaining to more specialist, or general functions, measured by the number of genes represented, from each network. Here, a positive value represents relative enrichment of GO terms of the given size, while a negative value represents relative lack of GO terms of the given size. (ii) Density plots E, F, G and H show overall relationship between the number of genes represented by the GO term (x-axis) and the maximum accuracy with which the term is captured by subgraphs from each network, measured using MCC (y-axis). Regions with denser shading indicate a greater number of GO terms.
(EPS)

File S1 **The PPI interaction network listed as pairwise interactions between nodes.** The first line of the file reports the number of edges in the network. This file also contains a lookup between node identifiers and the yeast systematic name reported in the SGD.
(ZIP)

File S2 **The genetic interaction network listed as pairwise interactions between nodes.** The first line of the file reports the number of edges in the network.
(ZIP)

File S3 **The coregulation interaction network listed as pairwise interactions between nodes.** The first line of the file reports the number of edges in the network.
(ZIP)

File S4 **The combined interaction network listed as pairwise interactions between nodes.** The first line of the file reports the number of edges in the network.
(ZIP)

File S5 **The 20 most accurately represented GO terms from each network and ontology.**
(XLS)
